# Potential negative consequences of geoengineering on crop production: A study of Indian groundnut

**DOI:** 10.1002/2016GL071209

**Published:** 2016-11-19

**Authors:** Huiyi Yang, Steven Dobbie, Julian Ramirez‐Villegas, Kuishuang Feng, Andrew J. Challinor, Bing Chen, Yao Gao, Lindsay Lee, Yan Yin, Laixiang Sun, James Watson, Ann‐Kristin Koehler, Tingting Fan, Sat Ghosh

**Affiliations:** ^1^ CMA Key Laboratory for Aerosol‐Cloud‐PrecipitationNanjing University of Information Science and TechnologyNanjingChina; ^2^ ICASSchool of Earth and Environment, University of LeedsLeedsUK; ^3^ International Center for Tropical AgricultureCaliColombia; ^4^ CGIAR Research Program on Climate ChangeAgriculture and Food SecurityCaliColombia; ^5^ Department of Geographical SciencesUniversity of MarylandCollege ParkMarylandUSA; ^6^ State Key Laboratory of Remote Sensing ScienceInstitute of Remote Sensing and Digital Earth, Chinese Academy of SciencesBeijingChina; ^7^ Climate Research UnitFinnish Meteorological InstituteHelsinkiFinland; ^8^ Department of Financial and Management StudiesUniversity of LondonLondonUK; ^9^ International Institute for Applied Systems AnalysisViennaAustria; ^10^ Queensland Alliance for Agriculture and Food InnovationUniversity of QueenslandBrisbaneQueenslandAustralia; ^11^ National Marine Environmental Forecasting CenterBeijingChina; ^12^ School of Mechanical and Building ScienceVellore Institute of TechnologyVelloreIndia

**Keywords:** geoengineering, agriculture, climate change, groundnut, GLAM

## Abstract

Geoengineering has been proposed to stabilize global temperature, but its impacts on crop production and stability are not fully understood. A few case studies suggest that certain crops are likely to benefit from solar dimming geoengineering, yet we show that geoengineering is projected to have detrimental effects for groundnut. Using an ensemble of crop‐climate model simulations, we illustrate that groundnut yields in India undergo a statistically significant decrease of up to 20% as a result of solar dimming geoengineering relative to RCP4.5. It is somewhat reassuring, however, to find that after a sustained period of 50 years of geoengineering crop yields return to the nongeoengineered values within a few years once the intervention is ceased.

## Key Points

- We simulated a negative impact on groundnut yields under geoengineered climate compared to a future global warming climate
- Crop yields are sensitive to water stress across whole Indian subcontinent, more sensitive to temperature stress across western part
- A sustained 50 year geoengineering intervention is shown to have no lasting effects on groundnut yields once the intervention is ceased

## 1 Introduction

According to the Intergovernmental Panel on Climate Change report [*Bindoff et al*., 2013], it is now virtually certain that the observed globally averaged temperature increase in the last 60 years is due mostly to humans. We are entering an unprecedented period of temperature change in recorded history and the scientific community has an important role to scientifically evaluate whether human intervention could possibly counteract anthropogenic climate change and the potential side effects. Geoengineering the climate, however, is a hugely complex area that also involves social, ethical, legal, and political issues [*Caldeira et al*., 2009]. There are many possible actions that are collectively known as geoengineering and range from diminishing the amount of the Sun's energy reaching the Earth's surface to directly capturing and storing greenhouse gases (GHGs) [*Robock*, 2008; *Robock et al*., 2008]. Numerical modeling results indicate that geoengineering may be able to moderate global mean temperature [*Govindasamy and Caldeira*, 2000; *Matthews and Caldeira*, 2007]; however, regional temperature changes will not necessarily respond in the same way as the global average [*Robock et al*., 2008]. There could be local swings in temperature and precipitation that could adversely affect yields and consequently the livelihoods of billions of people in developing countries [*Asseng et al*., 2014; *Challinor et al*., 2014b; *Porter et al*., 2014].

One of the geoengineering approaches that has received significant attention is the injection of sulphur dioxide into the lower stratosphere. The intention is that it would increase scattering of sunlight back to space and consequently reduce global temperature [*Robock*, 2008; *Jones et al*., 2011]. This solar dimming occurs periodically in nature as a consequence of volcanic eruptions and has been observed to successfully reduce global temperature and have no lasting effects a few years after the eruption. However, as solar dimming geoengineering does not directly lower GHGs then the global climate evolution will continue to be influenced by the elevated CO_2_ levels. This is likely to cause regional variations in temperature as well as impact on the biosphere [*Parmesan and Yohe*, 2003; *Challinor et al*., 2014b]. If regional temperatures or precipitation levels undergo significant deviations, either positive or negative, then this would affect the water cycle and all its dependencies [*Bala et al*., 2008; *Pongratz et al*., 2012; *Tilmes et al*., 2013]. We also need to recognize that entering into the uncharted climatic territory of geoengineering requires enhanced understanding of any possible biophysical and socioeconomic consequences before any action is taken. If geoengineering is to be viable, then of prime importance, in addition to lowering the global average temperature, is that it should not adversely affect the health, food supply, and livelihoods of the living population.

As with climate change scenarios, one of the main concerns with geoengineering is the possible impact on agriculture, as crop and livestock production systems are strongly dependent on weather and climate. A primary concern would be any potential effects on large human populations that depend on local crops or livestock for food or income, especially in rainfed systems, which is often the case for developing countries [*Mueller et al*., 2012]. With a world population expected to reach 9.6 billion by 2050 [*United Nations*, 2013], there are already increasing pressures on food supplies [*Foley et al*., 2011; *Lobell et al*., 2011]. Thus, we need to ensure that geoengineering does not exacerbate the current situation. There are significant potential risks of implementing geoengineering for the water cycle [*Bala et al*., 2008], and hence agriculture, that need to be thoroughly assessed with as many research tools as possible.

Our current understanding of geoengineering effects on crop production is far from comprehensive. To the knowledge of the authors, only three studies have investigated geoengineering impacts on crop productivity. *Pongratz et al*. [2012] assessed Solar Radiation Management (SRM) geoengineering impacts on maize, rice, and wheat production. Their results suggest increases in the global mean yields for all of these crops under SRM. For India, their results indicate decreasing maize yields and increasing wheat and rice yields. *Xia et al*. [2014] analyzed rice and maize yields over China by using the Decision Support System for Agrotechnology Transfer (DSSAT) crop model driven by output of 10 global climate models (GCMs) participating in the Geoegineering Model Intercomparison Project (GeoMIP). Consistent with *Pongratz et al*. [2012], they report minimal impact on rice and an increase of maize productivity when comparing results of geoengineered to nongeoengineered climate scenarios. The most recent study is that of *Parkes et al*. [2015], which assessed crop failures in a Marine Cloud Brightening (MCB) geoengineered climate scenario for Northeastern China and West Africa. They report reduced crop failures as a result of the geoengineering scheme.

We investigate the effects of solar dimming geoengineering for the groundnut crop for the Indian subcontinent and assess its impact on crop yields. India is one of the major groundnut producing countries in the world. It has the largest groundnut planting area of approximately 7.5 × 10^6^ ha and the second largest production, approximately 7 × 10^6^ metric ton annually [*Madhusudhana*, 2013; *Food and Agriculture Organization* (*FAO*), 2014]. In terms of the monetary significance, Indian groundnut is estimated to be about 3% of the agricultural output value [*Birthal et al*., 2014]. Our study seeks to add to the sparse body of literature on the effects of geoengineering on crops. This study analyses the effects of a sustained 50 year geoengineering intervention and, additionally, we evaluate the postgeoengineering period to determine if there are lasting changes that continue after the intervention has ceased.

## 2 Methods

### 2.1 Study Region

We focus on India because of its large population that is dependent on locally grown rainfed crops. The consequences of geoengineering will be important if significant changes in weather patterns result, especially if the onset or intensity of the Indian monsoon is affected [*Kravitz et al*., 2013; *Iles and Hegerl*, 2014]. We focus on the groundnut crop because of its financial importance for millions of poor smallholding farmers and farming communities [*Talawar*, 2004] and also because of its sensitivity to weather and climate variations [*Challinor et al*., 2004]. Additionally, India is one of the largest producer of groundnut globally [*FAO*, 2014]. In our modeling, we include all areas of India where groundnut is cultivated [*Ramirez‐Villegas et al*., 2015] and focus on the kharif (monsoon) season as groundnut production in this season comprises over 80% of total production [*Talawar*, 2004]. We divide India into five growing regions based on an existing agroecological analysis that accounts for soil factors, rainfall patterns, diseases, and pest patterns [*Talawar*, 2004] (supporting information Figure S1). According to the same study, germplasm characteristics within these regions are similar. We model all 2.8 × 2.8° grid cells (more on the climate model resolution in sections 2.2 and 2.3) where the average cultivated area in the period 1966–1993 is greater than 0.1%, amounting to 30 grid cells. In all aggregate yield calculations at the region level, the result presented is an average weighted by cultivated area.

### 2.2 Climate and Geoengineering Data

To study how geoengineering may influence groundnut yields we use output from climate model simulations from CMIP5 [*Andrews et al*., 2012; *Taylor et al*., 2012] for the future climate projections and GeoMIP [*Kravitz et al*., 2011] for the geoengineering projections. These output data were then used as inputs to run off‐line crop model simulations (see section 2.3). We focus on the BNU‐ESM climate model because (1) its simulations were one of only six models included in both the CMIP5 and G3 (the third GeoMIP experiment) GeoMIP intercomparisons, (2) according to previous research it had appropriate levels and realistic spatial patterns of precipitation for the summer Indian monsoon [*Sabeerali et al*., 2013], and (3) the model performed well or very well relative to other CMIP5 models for total precipitation, wet day frequency, mean temperature, and diurnal temperature range compared to historical observations (see supporting information Figure S2 and Text S2 [*Mitchell and Jones*, 2005; *Andrews et al*., 2012]). We chose to base our study on this one model that has been shown to reproduce appropriate spatial distributions of key meteorological parameters compared to observations rather than using a small ensemble that would include models with weaker performance in this region. Three scenarios were used in this work, historical and RCP (Representative Concentration Pathway) 4.5 scenarios from CMIP5 and G3 from GeoMIP. The historical (HIS) run in CMIP5 is from 1850 to 2005 and includes a historical time‐varying CO_2_ level. The future projection runs, RCP4.5 and G3, are initialized using this HIS run, starting in 2006, with a simulated increasing CO_2_ concentration. In the simulations, the CO_2_ concentration increases from 380 ppm in 2006 to 538 ppm by 2100. We chose RCP4.5 since it is a moderate climate change scenario compared to RCP 8.5 of CMIP5 business as usual trajectory that we are currently on [*Taylor et al*., 2012] and would therefore have some built‐in allowance for mitigation.

In the GeoMIP simulations, the geoengineering‐based injection of SO_2_ or sulphate aerosol into the lower stratosphere has begun at 2020 and shut off at 2069, and the simulation terminates at 2089 [*Kravitz et al*., 2011]. G3 is the third experiment of GeoMIP, which is designed to maintain RCP4.5 top of atmosphere net radiation at 2020 values by injection of sulphate aerosols, the sulphate aerosol is injected so that the forcing every year counteracts the anthropogenic GHG forcing [*Kravitz et al*., 2011].

### 2.3 Crop Model, Experiment Design, and Data Analysis

To estimate groundnut yields for geoengineered (G3) and nongeoengineered (RCP 4.5) scenarios, we used a regional‐scale process‐based crop model (the General Large Area Model for annual crops, GLAM) [*Challinor et al*., 2004] calibrated against historical yield observations [*Ramirez‐Villegas and Challinor*, 2016] and run off‐line using the meteorological and radiative data inputs from climate model simulations. In GLAM, crop growth and development are calculated for every modeled day depending on precipitation, mean, maximum, and minimum temperatures and downward shortwave solar radiation flux. Total biomass is calculated as the product of crop transpiration and a vapor pressure deficit (VPD)‐normalized transpiration efficiency, and yield is computed from the total crop biomass and a time‐integrated harvest index. For groundnut (a C3 crop), CO_2_ acts in GLAM to enhance water use efficiency and increase assimilation [*Challinor and Wheeler*, 2008]. The groundnut version of the GLAM model has been well tested in a variety of conditions, including for groundnut in India [*Challinor et al*., 2004; *Ramirez‐Villegas et al*., 2013; *Parkes et al*., 2015]. The model is described in more detail in *Challinor et al*. [2004] and *Challinor and Wheeler* [2008].

We performed GLAM crop simulations for the full duration of the climate runs, for each of the climate scenarios (HIS, RCP 4.5, and G3) at the original resolution of the BNU‐ESM climate model (i.e., 2.8 × 2.8°). This choice of resolution follows the definition of GLAM as a regional‐scale crop model and is consistent with previous GLAM studies, whereby GLAM is run at scales commensurate with those of climate models [*Challinor et al*., 2007; *Koehler et al*., 2013; *Parkes et al*., 2015; *Ramirez‐Villegas and Challinor*, 2016]. We note that downscaling of the climate model output was not needed. We used an ensemble of model parameters for GLAM derived from a previous study [*Ramirez‐Villegas and Challinor*, 2016]. Briefly, the authors developed a GLAM parameter ensemble by sampling the GLAM parameter space (20 parameters) and optimizing the model‐simulated yield against gridded district level yield and meteorological observations (see supporting information Text S1 for further details). This parameter ensemble, which consists of 19 independent parameter sets for the historical climate and 76 parameter sets for future simulations (19 × 4 CO_2_ response parameterizations) for each of the five groundnut growing regions (see supporting information Figure S1), were used for all simulations. For each parameter set and grid cell, we calibrated the yield gap parameter (YGP, varying between 0 and 1) [*Challinor et al*., 2004; *Ramirez‐Villegas and Challinor*, 2016]. Calibration of YGP is performed to (1) bias correct the climate model output and (2) to account for nonmodeled processes such as pest and diseases and fertilizers [*Challinor et al*., 2007, 2010]. For each grid cell and parameter set, the YGP calibration was determined by iteratively running the model with YGP values between 0 and 1 (in steps of 0.05) and then selecting the YGP value that minimized the perfect‐correlation mean squared error (PMSE) between model and observations for the historical period (1966–1993). Supporting information Text S1 provides more details of the GLAM model, the parameter ensemble, GLAM calibration procedures, CO_2_ response, and simulation configuration [*Jones et al*., 2003; *Challinor et al*., 2005a, 2006].

Using the calibrated parameter ensemble, we first performed all scenario model runs using the standard version of GLAM. Then, in order to understand the driving processes in the future scenarios, we also ran simulations with versions in which heat stress at the time of flowering was switched off (NOHTS), water stress was switched off (IRR), and both heat and water stress were switched off (IRR NOHTS). We then chose to focus on two future time periods both of 20 years' duration with the earlier period when geoengineering was active, from 2050 to 2069, and the second period when geoengineering was switched off, from 2080 to 2099. By studying these two time periods, we could then evaluate the effect of geoengineering on crops yields and determine if effects persisted after geoengineering was no longer active. The significance of changes in meteorology and yields were quantified using a statistical analysis approach including both the student *t* test and two‐sided Kolmogorov‐Smirnov tests for the modeled yield data for the scenarios.

## 3 Results

**Figure 1:**
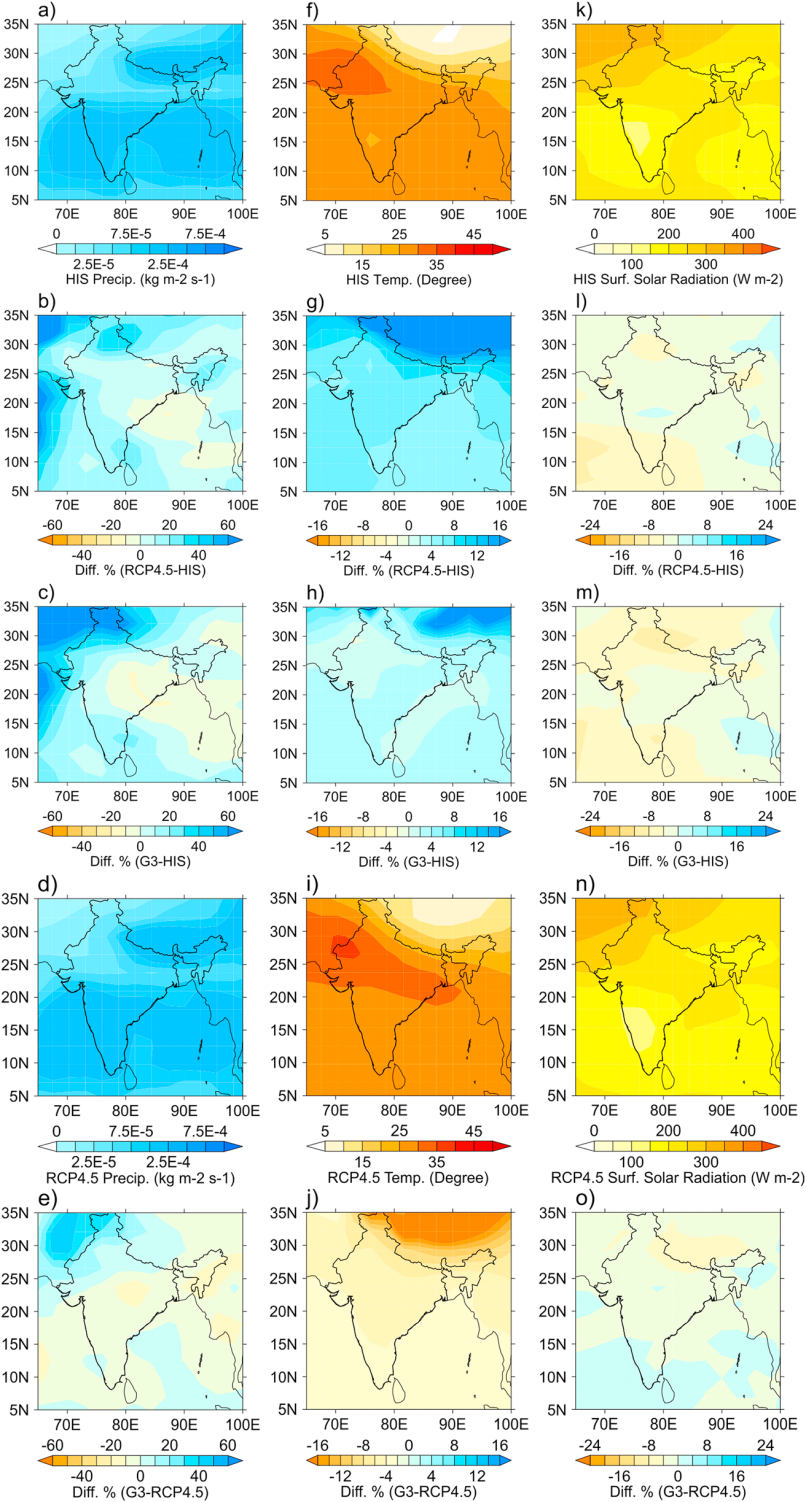
Simulated changes in climate for the HIS, RCP4.5, and G3 scenarios over the 2050–2069 period. An average over summer monsoon of (a–e) precipitation, (f–j) maximum temperature, and (k–o) solar flux at the surface for historical simulations (HIS, Figures 1a, 1f, and 1k), percentage difference of RCP4.5 and HIS (Figures 1b, 1g, and 1l), percentage difference of G3 and HIS (Figures 1c, 1h, and 1m), RCP4.5 (Figures 1d, 1i, and 1n), and percentage difference of RCP4.5 and G3 (Figures 1e, 1j, and 1n) for the time period 2050–2069 which is when geoengineering is active.

We use BNU‐ESM climate model projections from the CMIP5 framework to project the future climate and also to run the off‐line GLAM crop model. We use the RCP 4.5 which is a reference scenario for G3. The RCP4.5 is also a conservative assumption that is consistent with relatively ambitious emission reductions. We chose this value rather than the higher RCP 8.5 to build into the results scope for unforeseen future offsets by mitigation and adaptation. The BNU‐ESM for the RCP 4.5 pathway shows an increase in maximum temperature across all India throughout our simulation period (Figure 1 and supporting information Figure S3) which is consistent with what is expected for the global mean temperature with global warming. In general, the regional meteorology shows a clear response when geoengineering is active (Figure 1) that is clearly different compared to the results of RCP4.5 and HIS. We noted, however, that once geoengineering is shut off (supporting information Figure S3) the differences between RCP4.5 and G3 become negligible. Comparing G3 relative to RCP4.5 in Figure 1j, we see that the geoengineered climate is projected to result in a reduction of temperature, with significant decreases over the whole of India and with strong reductions in the northeast (NE) and into Nepal and China. In the supporting information Figure S3j, when geoengineering is switched off, it is shown that there are insignificant temperature differences between G3 and RCP4.5, indicating both simulations have converged toward a similar regional climate evolution.

For both the RCP 4.5 and G3 future scenarios, precipitation changes (compared to historical, HIS) are more geographically variable with negative changes in central and part of western India and increases elsewhere (Figures 1b and 1c). Once geoengineering is switched off (years 2080–2099; see supporting information Figures S3b and S3c), central and part of western India are seen to increase in precipitation, whereas the other regions decrease relative to when geoengineering was active. We find in general that switching off geoengineering makes the regional precipitation tend toward the nongeoengineered RCP 4.5 evolution. We find similar results for the modeled downward solar flux at the surface. It is reduced for almost all regions of India when geoengineering is active (2050–2069), with some small regions where it increases which we attribute to changes in cloud cover, and similarly tends toward the RCP 4.5 evolution once geoengineering is switched off.

**Figure 2:**
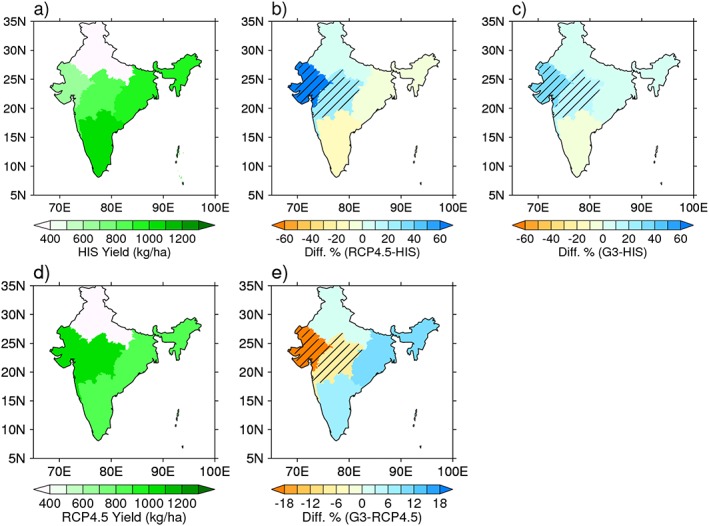
Projected yield changes for HIS, RCP4.5, and G3 scenarios over 2050–2069 period. Groundnut crop yields for India for when geoengineering is active, time period 2050–2069. Shown are (a) yields for HIS, (b) percentage difference of RCP and HIS, (c) percentage difference of G3 and HIS, (d) RCP, and (e) percentage difference of RCP and G3. The hatched regions indicate that there is a statistically significant difference between the yields of the two scenarios being compared, according to the Kolmogorov‐Smirnov (KS) and student's *t* tests.

**Figure 3:**
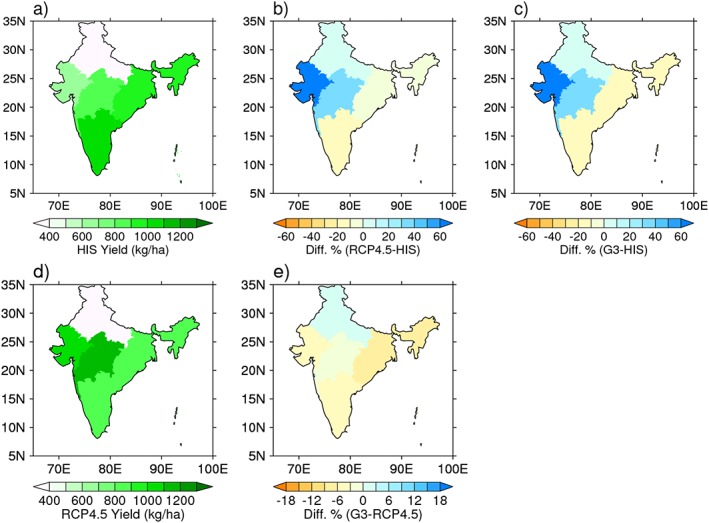
Yield changes for HIS, RCP4.5, and G3 scenarios over 2080–2099 period. Groundnut crop yields for India for when geoengineering is inactive, time period 2080–2099. Shown are yields for (a) HIS, (b) percentage difference of RCP and HIS, (c) percentage difference of G3 and HIS, (d) RCP, and (e) percentage difference of RCP and G3.

**Figure 4:**
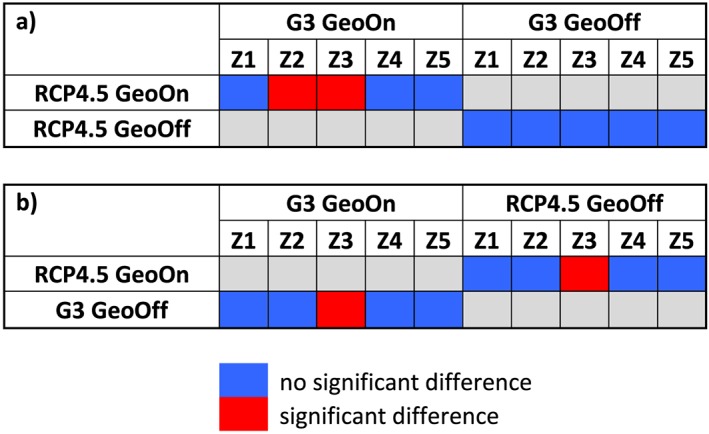
The top part indicates the statistical test between RCP4.5 and G3 for both 2050–2069 (GeoOn) and 2081–2099 (GeoOff). The bottom part indicates the statistical tests between GeoOn and GeoOff for each scenario (e.g., RCP4.5 and G3). Blue indicates that there is no statistically significant difference between two scenarios for that region, and red indicates that there is statistically significant difference. Grey indicates that the statistical test for comparison was not performed.

The simulated future scenarios were used as inputs into GLAM, and the results are shown in Figures 2 and 3. It is apparent that for the historical period that North and South India have opposite responses for the RCP 4.5 and G3 scenarios. While the north shows increasing yields, the south shows slightly decreasing yields. In the north, the yields are sensitive to precipitation changes and yields increase, sometimes substantially, for all future scenarios relative to historical runs (supporting information Figure S4). We see that for both RCP4.5 and G3 future climate projections the yields relative to historical are projected to rise for the regions approximately 17°N to 27°N for western, northern, and central India (regions 1–3, Figures 2b and 2c). From these, only western and central India show statistically significant changes in yields for G3 relative to RCP 4.5 (2050–2069) (Figure 4).

GLAM is a process‐based model, so we are able to understand the driving factors underlying the regional changes. Unlike in the south, where there is generally sufficient precipitation, Northern India crops are very sensitive to precipitation changes and we see differences in the precipitation in the NE compared to the northwest (NW). This is also seen by the large yield gains from increased water supply (as seen in supporting information Figure S4). When geoengineering is active (2050–2069), we note that the NW shows an increase in precipitation for G3 relative to both RCP 4.5 and HIS, whereas for the NE the opposite is true (Figures 1b, 1c, and 1e). In the NW region the mean temperature is higher than the optimal temperature for groundnut crop development (*T*_O_ = 28°C) and so the projected temperature decreases and results in a yield reduction mediated through accelerated development (and hence shorter stages of development). The projected reductions in solar flux also acts to reduce yields through reduced light interception by the plants (Figures 1l, 1m, and 1o and supporting information Figures S3l, S3m, and S1o). Thus, for the NW, which is very precipitation sensitive, the increases in precipitation are deemed responsible for the increase in yield in both RCP 4.5 and G3 relative to the historical period in addition to the effects of increased CO_2_ [*Challinor and Wheeler*, 2008]. Since the CO_2_ levels are the same in G3 and RCP4.5, both mean temperature and precipitation are deemed responsible for the yield decreases in G3 relative to RCP4.5. We note that in the NW the heat stress around flowering is an important factor for both G3 and RCP (supporting information Figure S4, second column).

In central India (region 3), the precipitation is slightly decreased for G3 (compared to HIS, see Figure 1c) so precipitation cannot explain the projected increases in yields for G3 relative to HIS. Additionally, mean temperature in the G3 scenario is near HIS levels; hence, the slight temperature reductions in parts of central India in G3 compared to HIS alone cannot account for the yield changes. The remaining factor is CO_2_, which acts to increase the groundnut yield and could indeed account for the observed gains [*Challinor and Wheeler*, 2008]. A reduced percentage yield for central India compared to NW for G3 relative to RCP 4.5 is due to the counteracting influence of the temperature being lower than the optimal growth temperature, resulting in a higher yield, and lower yield from a reduced precipitation. Also, in relative terms, reduced precipitation in the NW, which is a more precipitation‐sensitive area, impacts crop yields more substantially than the precipitation reductions in central India. Mean temperature and precipitation are therefore important factors for the changes observed in central India. Furthermore, Figure 3 indicates that once geoengineering is switched off, yields for RCP4.5 and G3 show no appreciable differences. Both RCP 4.5 and G3 scenarios, however, show significant increases in yields relative to historical values especially for the NW and central India.

## 4 Discussion

Many previous works using statistical or process‐based crop modeling show that global warming climate will have significant negative effects on crop yields [*Wheeler et al*., 2000; *Auffhammer et al*., 2006]. Our projections for groundnut yields in India suggest there will be increases with future climate change. Our projections are consistent with past works of groundnut [*Challinor et al*., 2007; *Ramirez‐Villegas and Challinor*, 2016] that show increasing yields for Western India, but we note differences for central India, where we predict a statistically significant increase and past studies predict a decrease. Although we used the same crop model, there were several differences in the studies that can account for this difference. The past works not only used a much shorter time horizon (only to 2030s), so there was little temporal overlap, but they also used a different GCM to derive the inputs for the crop model. We have confidence that the BNU‐ESM model used in our study was optimal for this work based on its ability to reproduce realistic historical spatial patterns of temperature and precipitation (see below for further discussion). Finally, this study only attributed changes if the yields showed statistical significance using both the student's *t* test and Kolmogorov‐Smirnov test, which is why we only note the yields of two regions showed changes.

The literature to date addressing the effects of geoengineering on crops is sparse, and only a few types of crops have been considered but not groundnut. In terms of the global mean yields for wheat, maize and rice, results all show an increase relative to the yields based on future climate change [*Pongratz et al*., 2012]. A study focusing on China [*Xia et al*., 2014] also showed increasing yields except for rice, which showed a decrease but was noted to be consistent with *Pongratz et al*. [2012] when only considering China. Our results for groundnut show a decreased yield for geoengineering relative to the global warming scenario which is opposite to most of the results in the literature above. We attribute the decrease in the central area to originate from a reduction of precipitation (down 2%) compared to the RCP4.5 simulations (which is also evident in the ensemble mean results).

We highlight that this study uses the same irrigation use/availability in all future scenarios. However, research indicates that the mean water table is significantly decreasing, ranging from 0.3 m to 1 m annually across India [*Hira*, 2009]. For example, predictions indicate that the water table depth will increase from 22.8 m in 2006 to 42.5 m by 2023 for central Punjab [*Hira*, 2009]. The surface water resources are also shrinking [*Mall et al*., 2006] especially for snow‐dominated regions (e.g., runoff rivers from the Himalayas), as warmer temperature are causing alterations to the supply of the water that affect the crop growing season [*Barnett et al*., 2005]. All of these factors are likely to have some effect on future groundnut yields and so could be included in future studies.

Lastly, we acknowledge that climate‐based modeling research is subject to a number of uncertainties [see *Challinor et al*., 2014b, 2014a], most notably the climate and crop models. Our choice of climate model was restricted to those that had participated in both the CMIP and GeoMIP intercomparisons. From those models, the BNU‐ESM was reported to have realistic mean and spatial patterns of relevant crop variables (temperature, diurnal range, precipitation, wet day frequency, etc.) for India relative to observations and was also in agreement with the CMIP5 multimodel ensemble results [*Knutti and Sedláček*, 2012] (see supporting information Figure S2). We selected the GLAM crop model since it was previously used to study groundnut in India [*Challinor et al*., 2005b; *Ramirez‐Villegas and Challinor*, 2016] and is a large‐scale process‐based model appropriate for modeling domains the size of India at climate model resolutions.

## 5 Conclusions

This study made projections about the effects of solar dimming geoengineering for Indian groundnut crop yields. Groundnut is an important cash crop in India since it is linked with the livelihood of millions of people and so it is important to assess potential geoengineering impacts for this crop. We performed process‐based crop modeling based on projections of geoengineering and RCP 4.5 climate change using the BNU‐ESM climate model, which was used in both CMIP and GeoMIP. Our results show that solar dimming geoengineering would significantly affect groundnut crop yields for two large regions of India (western and central regions) resulting in reduced yields of up to 20% relative to the RCP4.5 scenario. The results for the west and central regions were shown to be statistically significant, whereas the yields for other regions of India were not. Groundnut yields were shown to be sensitive to regional variations in meteorological variables, and our process simulations showed significant importance of water stress for all India, whereas heat stress was found to be important only for the west. Although we have shown that geoengineering can have significant negative effects on yields, we have also shown that once geoengineering is switched off, even after a continuous 50 year period, the yields tended toward the values of the nongeoengineered climate change scenario (RCP) with no statistical difference. This is important as it statistically shows that a sustained period of geoengineering intervention is not likely to set us on a vastly different climatic path and that the lower groundnut crop yields for geoengineered climate would rebound once geoengineering was ceased.

## Supporting information



Supporting Information S1

Figure S1

Figure S2

Figure S3

Figure S4
