# Supplementary material for: Potential negative consequences of geoengineering on crop production: A study of Indian groundnut
Source: Geophys Res Lett. 2016 Nov 19;43(22):11786–95. doi: 10.1002/2016GL071209 (PMC5267972; doi:10.1002/2016GL071209)
Supplement: Supplementary file 1 — Supporting Information S1 [file GRL-43-11786-s001.docx]

**Supplementary Information**

**Title**

Potential negative consequences of geoengineering on crop production: a study of Indian groundnut

**Authors**

Huiyi Yang^1,2 *^, Steven Dobbie^1^, Julian Ramirez-Villegas^1,3,4^, Kuishuang Feng^5^, Andrew J. Challinor^1,4^, Bing Chen^6^, Yao Gao^7^, Lindsay Lee^1^, Yan Yin^2^, Laixiang Sun^5,8,9^, James Watson^10^, Ann-Kristin Koehler^1^, Tingting Fan^11^ and Sat Ghosh^12^

**Affiliations**

^1^ ICAS, School of Earth and Environment, University of Leeds, Leeds, LS2 9JT.

^2^ CMA Key Laboratory for Aerosol-cloud-precipitation, Nanjing University of Information Science & Technology, Nanjing, China.

^3^ International Center for Tropical Agriculture, Cali, Colombia.

^4^ CGIAR Research Program on Climate Change, Agriculture and Food Security, Cali, Colombia.

^5^ Department of Geographical Sciences, University of Maryland, College Park, USA

^6^ The State Key Laboratory of Remote Sensing Science, Institute of Remote Sensing and Digital Earth, Chinese Academy of Sciences, Beijing 100101, China

^7^ Climate Research Unit, Finnish Meteorological Institute, P.O. Box 503, Helsinki, Finland

^8^ Department of Financial & Management Studies, University of London, London, UK

^9^ International Institute for Applied Systems Analysis (IIASA), A-2361 Laxenburg, Austria

^10^ University of Queensland, Brisbane QLD 4072, Australia

^11^ National Marine Environmental Forecasting Center, Beijing, China.

^12^ Vellore Institute of Technology, Vellore, Tamil Nadu, India. 632 014.

*Corresponding author: Huiyi Yang ([h.yang@leeds.ac.uk)](mailto:h.yang@address.edu))

**Contents of this file**

1. Text S1 GLAM model description, model calibration and simulation configuration
2. Text S2 Overview of BNU-ESM model simulations
3. Figure S1 Study regions
4. Figure S2 Skill of CMIP5 models to simulate monsoon climatology.
5. Figure S3 Simulated climate changes for HIS, RCP4.5 and G3 scenarios over the 2081-2100 period.
6. Figure S4 Impact of water and heat stress.

**Text S1: GLAM model description, model calibration and simulation configuration**

To investigate potential impacts of RCP4.5 climate change and geoengineering scenarios on groundnut crop yields, we used the General Large Area Model for annual crops (GLAM) (Challinor et al. 2004). GLAM was specifically designed for the scales we focus on in this study and has been used extensively for the Indian region [*Challinor et al.,* 2004, 2005, 2006; *Ramirez-Villegas et al.,* 2015; *Ramirez-Villegas and Challinor,* 2016]. It is a regional-scale process-based model specifically designed to capture the non-linear relationships between climate and crop growth and, hence, is of relatively lower complexity compared to site-specific models designed for decision support at field scales (e.g. *Jones et al.* [2003]). In the groundnut version of GLAM, crop development is calculated on a daily growth timescale using a triangular function to describe the rate of development with three cardinal temperatures. Five plant stages are defined for the groundnut development: planting to emergence (PL-EM), emergence to flowering (EM-FL), flowering to pod initiation (FL-PF), pod initiation to maximum leaf area index (PF-LMAX), and maximum leaf area index to physiological maturity (LMAX-PM). Changes from one stage to the next are triggered by the exceedance of crop phasic thermal requirements. In all simulations, unless otherwise stated, harvest occurs at physiological maturity.

GLAM inputs include daily weather, soil type, hydrological properties, planting date, and crop- and region-specific growth parameter values and a site-specific yield gap parameter (YGP) (see below and *Challinor et al.,* [2004]). GLAM explicitly models the crop growth on a daily basis based on weather, sunlight, soil properties, seed types and planting dates as inputs as well as accounting for a range of other factors as well (i.e. pests, diseases, nutrition, and management practices) through a yield gap parameter (YGP, which varies between 0 and 1). To some extent, GLAM’s YGP can also account for errors in input data (particularly when climate model simulations are used) [*Challinor et al.*, 2004, 2006]. In GLAM, YGP is used to reduce leaf area index (LAI), which is calculated using a parameterized daily rate of change reduced by water stress. Daily crop biomass is computed as the product of the crop’s daily transpiration and a normalized (by vapour pressure deficit, VPD) transpiration efficiency. Daily evapotranspiration is calculated after accounting for energetic (through the Priestley-Taylor equation), physiological (leaf area) and water availability (soil moisture) limitations, and partitioned to evaporation and transpiration depending on whether the potential environmental demand is satisfied or not. Crop yield in the model is calculated as the product of the total biomass and a time-integrated harvest index. The rate of change in the harvest index is a model parameter. Drought and heat stress around flowering and terminal drought stress are also included in GLAM as a way of simulating the impact of extreme sub-seasonal conditions on crop growth (see *Challinor et al.*, [2006]).

In order to calibrate GLAM, the definition of 23 site-independent (‘global’) and one site-specific (‘local’) model parameters (i.e. the YGP) are required [*Challinor et al.*, 2004; *Ramirez-Villegas and Challinor*, 2016]. We use global parameters that are defined separately for each of the regions shown in Supplementary Fig. S1 (defined after *Talawar* [2004]), whereas YGP is defined for each grid cell in the analysis domain. Grid cells where crop model runs were conducted were those in which the proportion of area used for groundnut growing was 0.2% or larger [*Ramirez-Villegas et al.,* 2015; *Ramirez-Villegas and Challinor,* 2016]. Because only yield observations were available to us for model calibration, we used a parameter ensemble that allowed for the effect of parametric uncertainty in all our model simulations, i.e. historical (HIS), RCP4.5, and geoengineering (G3) scenarios. The development of the parameter ensemble is described elsewhere (see *Ramirez-Villegas and Challinor,* [2016]), and hence a full description is not provided here. Briefly, an initial total of 50 parameter sets was developed for each of the growing zones in Supplementary Fig. S1 based on observed yields and meteorological data using the root mean squared error (RMSE) as the measure of skill. Variation in the final parameter values for each parameter sets was achieved by altering the starting point of the parameter space and the order of parameter calibration. Finally, from the 50 initial parameter sets, the one with the lowest RMSE (reference) was selected and compared with the remaining 49 using a Kolmogorov-Smirnov non-parametric test. For each growing zone, this yielded 19 final parameter sets whose distribution was found statistically similar to the reference. *Ramirez-Villegas and Challinor* [2016] provide a comprehensive analysis of the skill of the 19-member GLAM parameter ensemble used here.

For each of the 19 parameter sets in each growing zone, YGP was then calibrated on a grid cell basis using the HIS climate simulations of the BNU-ESM global climate model. To find the optimal YPG value (in the range of 0-1), we used the method of *Challinor et al.* [2007] where model skill and YGP calibration were determined using the perfect-correlation mean squared error (PMSE, Eq. 1).

$PMSE=\left( \bar{o}-\bar{s} \right)^{2}+\left( \sigma_{o}-\sigma_{s} \right)^{2}$ [1]

where $\bar{o}$ and $\bar{s}$ are the time-means (1966-1990) for observed and simulated yields, respectively, and *σ_o_* and *σ_s_* correspond to the standard deviations of yield observations and simulations. This method is based on the relationship between centred mean bias and the ratio of standard deviations between yield observations and simulations under the assumption of perfect correlation. Yield observations were obtained from previous GLAM studies [*Ramirez-Villegas et al.,* 2015; *Ramirez-Villegas and Challinor,* 2016; *Challinor et al.*, 2004].

Once YGP had been calibrated, we performed crop simulations for the full duration of the climate runs, for each of the climate scenarios (HIS, RCP4.5, G3), using historically (HIS) calibrated YGP values for future scenarios under the assumption that yield gaps and GCM meteorology bias is relatively constant in time. For the future climate scenarios, the simulation of crop response to elevated CO_2_ concentrations was included. Therefore, we used four CO_2_ response parameterizations based on *Challinor and Wheeler* [2008] and *Ramirez-Villegas and Challinor* (2016). Simulations were conducted with moderate and large increases in transpiration efficiency, in conjunction with decreases in the physiologically limited transpiration rate, a decrease in specific leaf area, and a moderate and low sensitivity of the crop to CO_2_ enhancement under low vapour pressure deficit (VPD) conditions. For additional details of simulation of CO_2_ response in GLAM, the reader is referred to *Challinor and Wheeler* [2008]. The simulation of the CO_2_ response for every location and future scenario (G3, RCP4.5) involved simulations for a total of 76 (19 baseline parameter sets x 4 CO_2_ response parameterizations) different parameter settings. This enabled the assessment of parameter uncertainty, CO_2_ response uncertainty and their interactions at the process-level, in this work.

For each scenario, we conducted four sets of simulations. The first set (default model version) included all biophysical processes and stresses so as to provide as close as possible a representation of groundnut crop growth, and hence results were used for all figures in the main paper. The remaining three sets of simulations were performed only to investigate the impact of specific processes, namely, heat stress at the time of anthesis (second set, NOHTS), fully removing water stress (third set, IRR), and both heat stress and water stress simultaneously (fourth set, IRR NOHTS). For the second set of simulations the heat stress at the time of anthesis was switched off in GLAM. The third set of simulations was conducted with the potential water balance instead of the actual water balance. The fourth set of runs was a combination of IRR and NOHTS and was run with the heat stress at anthesis turned off in the parameterization and with the potential water balance.

**Supplementary Text S2: Overview of BNU-ESM model skill and its representativeness in the CMIP5 model ensemble**

The Taylor diagram [*Taylor*, 2001] provides a statistical framework to graphically summarise how well model simulations compare to observations. The similarity of model simulation and observations is quantified in terms of the correlation coefficient, the Root Mean Square Error (*RMSE*) and the ratio of variances. In the Taylor diagram, a single point in a two-dimensional plot is used to indicate these three measures. The Taylor diagram is widely used to evaluate the performance of complex models such as IPCC climate models.

We used the Taylor diagrams developed by two previous studies for CMIP3 and CMIP5 models [*Ramirez-Villegas et al.,* 2013*; Ramirez-Villegas,* 2016] to analyse the performance of GCMs. These studies used the Climatic Research Unit (CRU) gridded historical observation-based dataset [*Mitchell and Jones.,* 2005]. Only the mean climate was analysed. Four key variables determining the crop growth were examined, namely, total precipitation, wet-day frequency, mean temperature and diurnal temperature range. Figure S2 shows the Taylor diagram for all these simulated variables. The plot is constituted by three axes. The *x*-axis is the ratio of standard deviations (between models and observations). The arcs concentric to the reference measure (marked with a hollow black circle on the *x*-axis) are the centred *RMSE* (normalised by the standard deviation of the observations). The azimuthal position indicates the correlation.

The Taylor diagram indicates that the BNU-ESM (identified by the red letter ‘W’ in Fig. S2) climate model is one of the best performing GCMs for the mean climate in South Asia. For the diurnal temperature range, in particular, it performs better than the multi-model mean (MMM), whereas for mean temperature and total precipitation it performs similarly to the MMM. For the wet-day frequency, in general, all GCMs and the MMM show poor performance. For this variable, the BNU-ESM model shows *RMSE* and standard deviation values that are comparable to those of the MMM, but its correlation is lower. Other studies also showing its spatial distribution of meteorological variables were appropriate [*Sabeerali* *et al* 2013]. Based on these results, we deem the BNU-ESM climate model suitable for our modelling analysis . We note, however, that whilst it was not within the objectives of our paper, using more GCMs would allow the quantification of the effect of the uncertainty in the climate response [*Andrews et al.,* 2012] on the simulated geoengineering impacts on the groundnut crop yields.


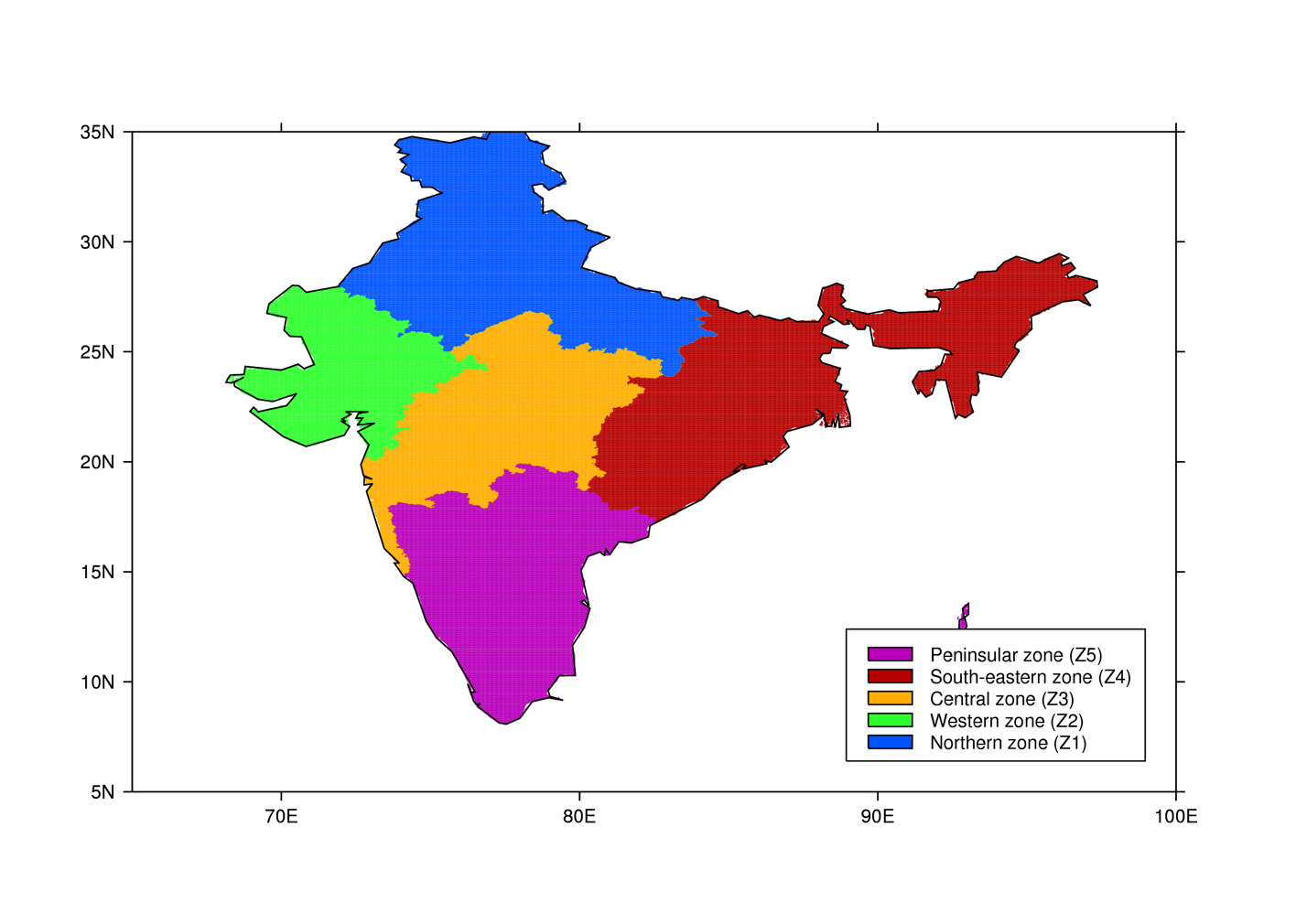


**Figure S1** Study region. This indicates the division into five groundnut growing zones. The regions were chosen based on climate, soil type, and cultivars, and follows *Talawar* [2004].


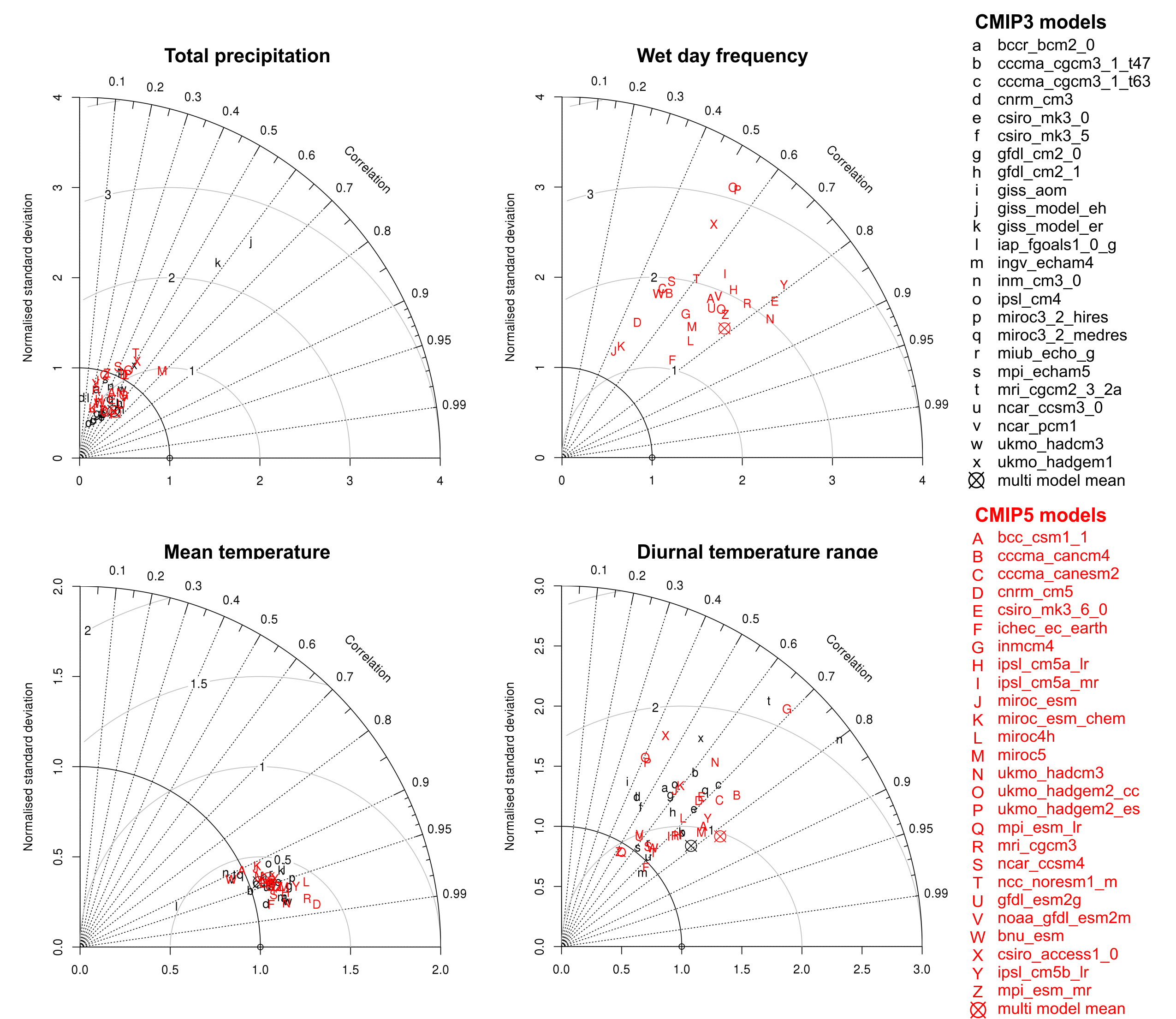


**Figure S2** Taylor diagrams for 23 CMIP3 (black lower case letters) and 26 CMIP5 (red upper case letters). Multi model means are indicated by crossed circles in the respective colour. Only climatological means of simulated annual totals (precipitation and wet-day frequency) and means (mean temperature and diurnal temperature range) for India are shown. In all cases only comparisons with CL-CRU are shown. Taken from *Ramirez-Villegas et al.*, [2013] and *Ramirez-Villegas* [2016].


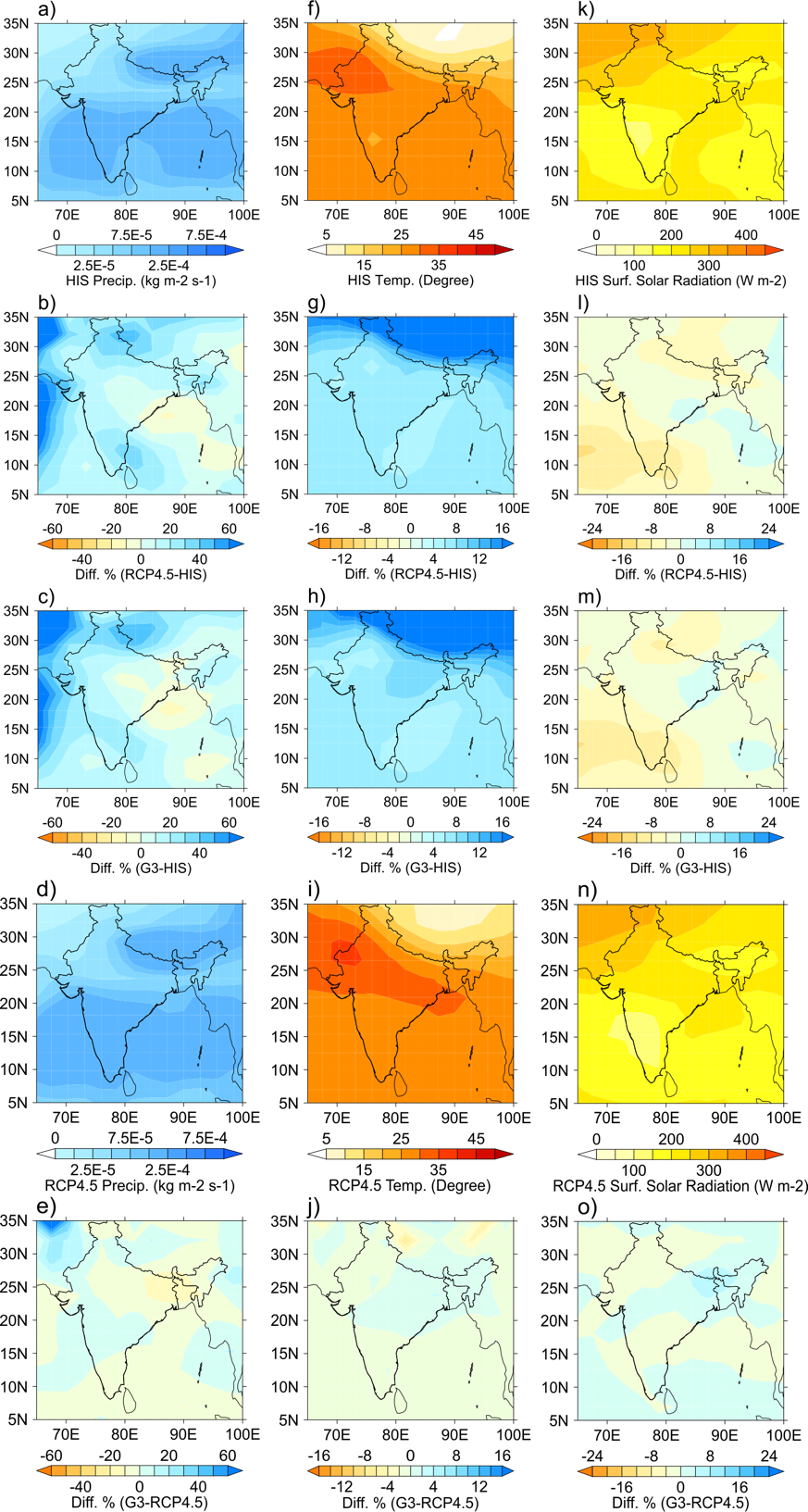


**Figure S3** Simulated changes in climate for the HIS, RCP4.5 and G3 scenarios over the 2080-2099 period. An average over June-July-August-September of precipitation (first column, panels a–e), maximum temperature (second column, panels f–j), and solar flux at the surface (third column, panels k–o) for historical simulations (HIS, first row, panels a, f, k), percentage difference of RCP4.5 and HIS (second row, panels b, g, l), percentage difference of G3 and HIS (third row, panels c, h, m), RCP4.5 (fourth row, panels d, i, n), and percentage difference of RCP4.5 and G3 (fifth row, panels e, j, n) for the time period 2080-2099 which is when geoengineering is off.


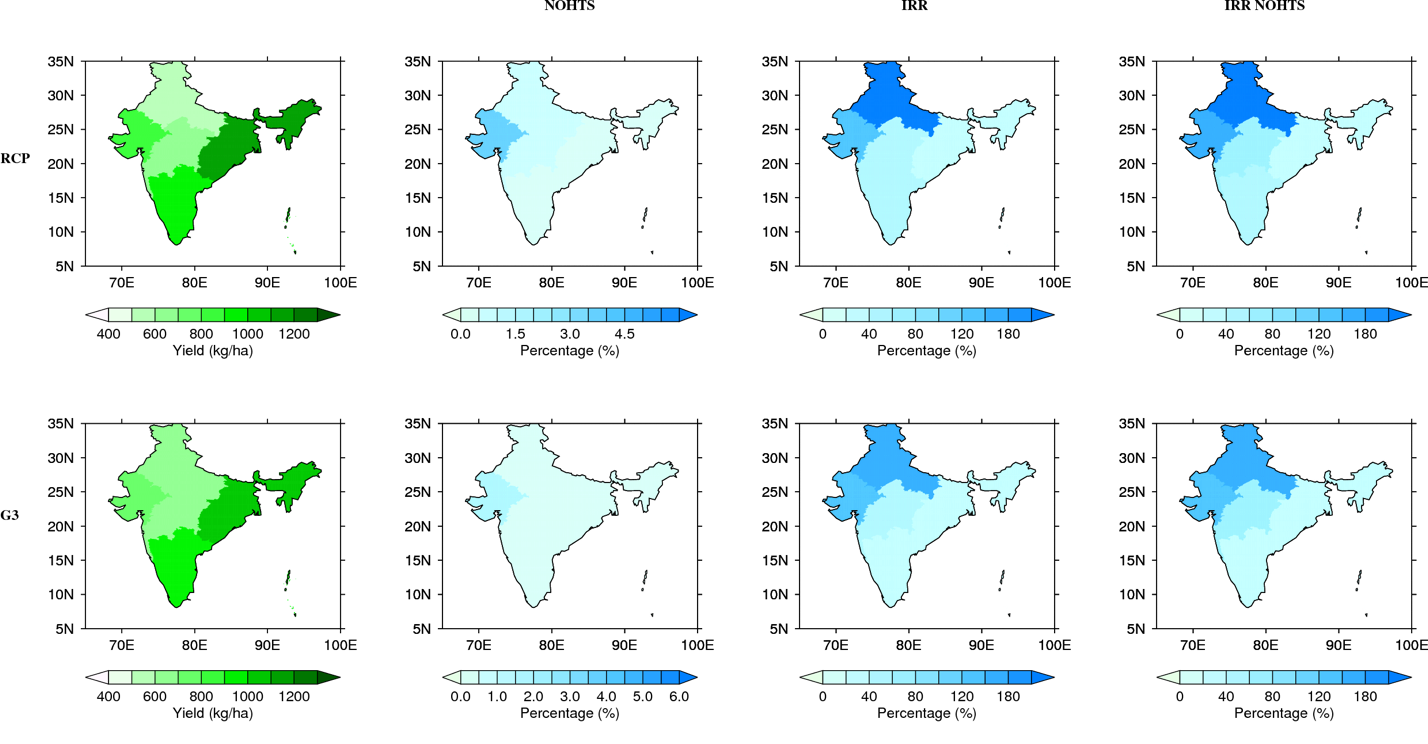


**Figure S4** Impact of water and heat stress. Results of groundnut yields (first column) for the different scenarios with effects of heat and water stresses. Relative change in yield for RCP and G3 scenarios for simulations without heat stress at flowering (NOHTS), with sufficient water supply (i.e. fully irrigated, IRR), and both without heat stress at the time of flowering and fully irrigated (IRR NOHTS).

**Supplementary References**

Andrews, T., J. M. Gregory, M. J. Webb, and K. E. Taylor (2012), Forcing, feedbacks and climate sensitivity in CMIP5 coupled atmosphere-ocean climate models, *Geophys. Res. Lett.*, *39*(9), L09712, doi:10.1029/2012gl051607.

Challinor, A. J., T. R. Wheeler, P. Q. Craufurd, J. M. Slingo, and D. I. F. Grimes (2004), Design and optimisation of a large-area process-based model for annual crops, *Agric. For. Meteorol.*, *124*(1-2), 99–120, doi:10.1016/j.agrformet.2004.01.002.

Challinor, A. J., J. M. Slingo, T. R. Wheeler, and F. J. Doblas–Reyes (2005), Probabilistic simulations of crop yield over western India using the DEMETER seasonal hindcast ensembles, *Tellus A*, *57*(3), 498–512, doi:10.1111/j.1600-0870.2005.00126.x.

Challinor, A. J., T. R. Wheeler, T. M. Osborne, and J. M. Slingo (2006), *Assessing the vulnerability of crop productivity to climate change thresholds using an integrated crop-climate model*, edited by J. Schellnhuber, W. Cramer, N. Nakicenovic, T. M. L. Wigley, and G. Yohe, Cambridge University Press, Cambridge, UK.

Challinor, A. J., T. R. Wheeler, P. Q. Craufurd, C. A. T. Ferro, and D. B. Stephenson (2007), Adaptation of crops to climate change through genotypic responses to mean and extreme temperatures, *Agric. Ecosyst. Environ.*, *119*(1-2), 190–204, doi:10.1016/j.agee.2006.07.009.

Challinor, A. J., and T. R. Wheeler (2008), Use of a crop model ensemble to quantify CO2 stimulation of water-stressed and well-watered crops, *Agric. For. Meteorol.*, *148*(6-7), 1062–1077, doi:10.1016/j.agrformet.2008.02.006.

Jones, J. W., G. Hoogenboom, C. H. Porter, K. J. Boote, W. Batchelor, L. A. Hunt, P. Wilkens, U. Singh, A. Gijsman, and J. Ritchie (2003), The DSSAT cropping system model, *Eur. J. Agron.*, *18*(3-4), 235–265, doi:10.1016/S1161-0301(02)00107-7.

Mitchell, T. D., and P. D. Jones (2005), An improved method of constructing a database of monthly climate observations and associated high-resolution grids, *Int. J. Climatol.*, *25*(6), 693–712, doi:10.1002/joc.1181.

Ramirez-Villegas, J., A. J. Challinor, P. K. Thornton, and A. Jarvis (2013), Implications of regional improvement in global climate models for agricultural impact research, *Environ. Res. Lett.*, *8*(2), 24018.

Ramirez-Villegas, J., A.-K. Koehler, and A. J. Challinor (2015), Assessing uncertainty and complexity in regional-scale crop model simulations, *Eur. J. Agron.*, doi:10.1016/j.eja.2015.11.021.

Ramirez-Villegas, J., and A. J. Challinor (2016), Towards a genotypic adaptation strategy for Indian groundnut cultivation using an ensemble of crop simulations, *Clim. Change*, 1–16, doi:10.1007/s10584-016-1717-y.

Sabeerali C T, Ramu Dandi A, Dhakate A, Salunke K, Mahapatra S and Rao S A (2013) Simulation of boreal summer intraseasonal oscillations in the latest CMIP5 coupled GCMs *J. Geophys. Res. Atmos.* **118** 4401–20 Online: http://doi.wiley.com/10.1002/jgrd.50403

Talawar, S. (2004), *Peanut in India: History, Production and Utilization*, University of Georgia, Athens, Georgia, USA.
