# Supplementary figures and images for: Potential negative consequences of geoengineering on crop production: A study of Indian groundnut
Source: Geophys Res Lett. 2016 Nov 19;43(22):11786–95. doi: 10.1002/2016GL071209 (PMC5267972; doi:10.1002/2016GL071209)

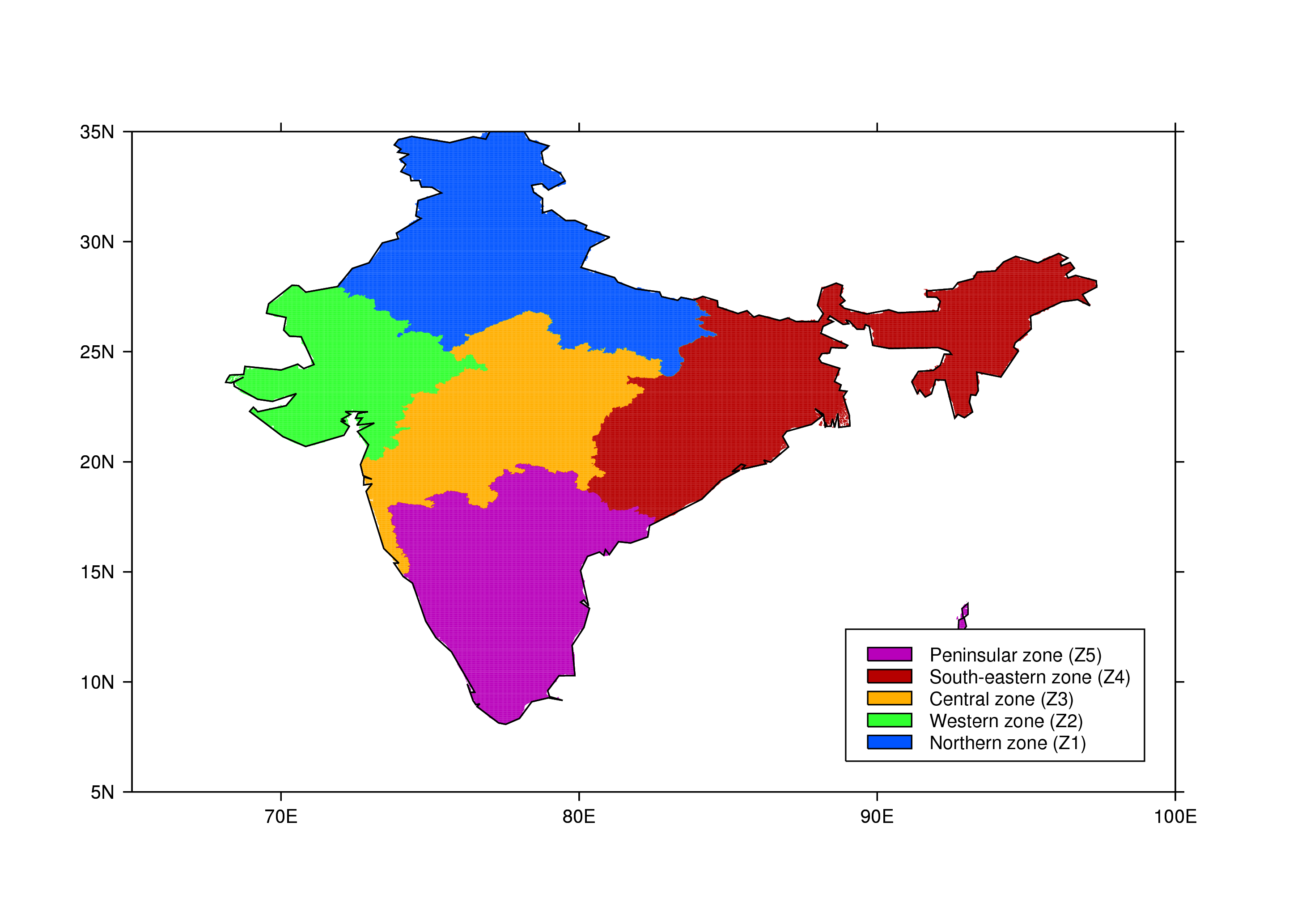

Supplement: Supplementary file 2 — Figure S1 [file GRL-43-11786-s002.png]

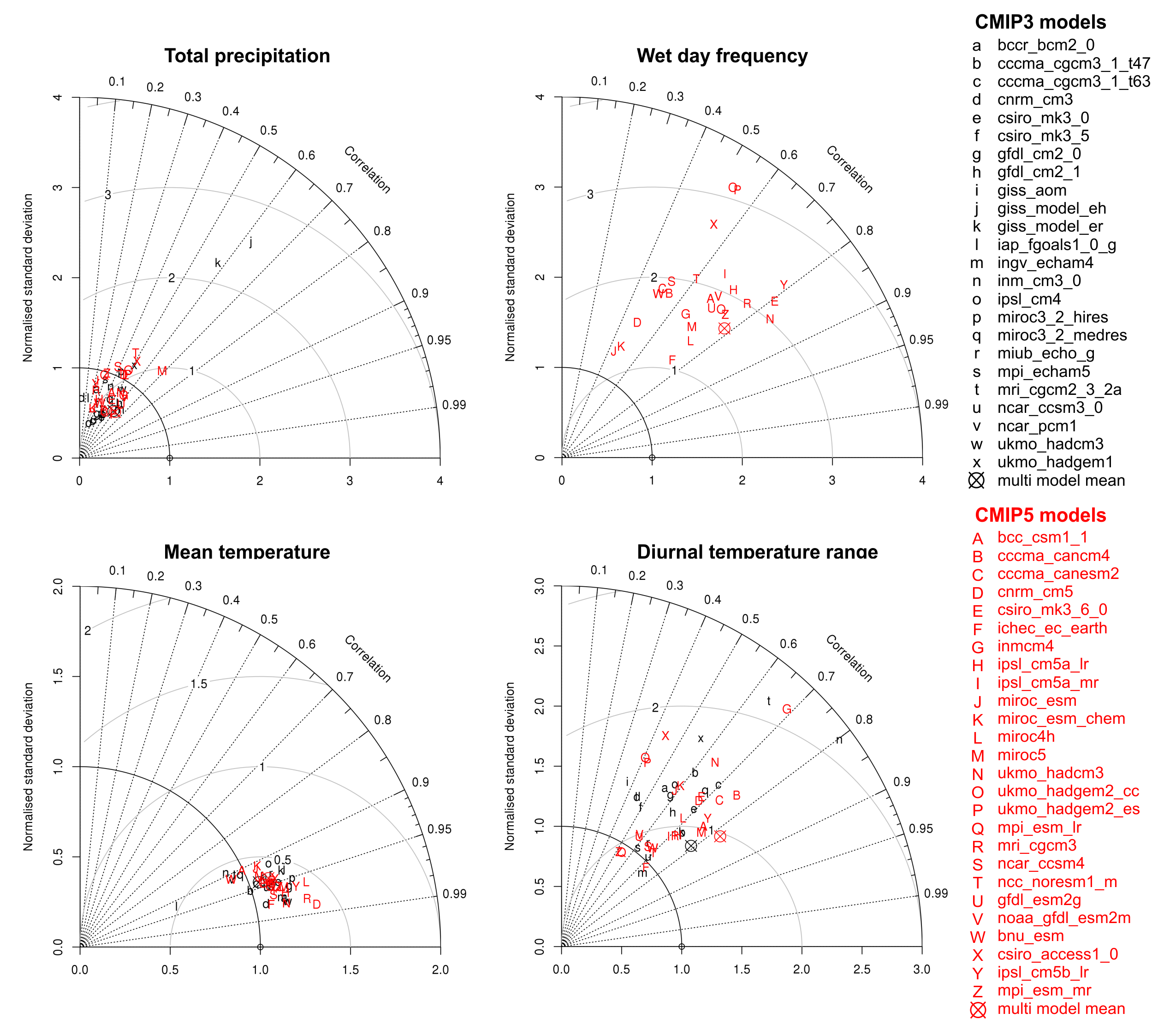

Supplement: Supplementary file 3 — Figure S2 [file GRL-43-11786-s003.tif]

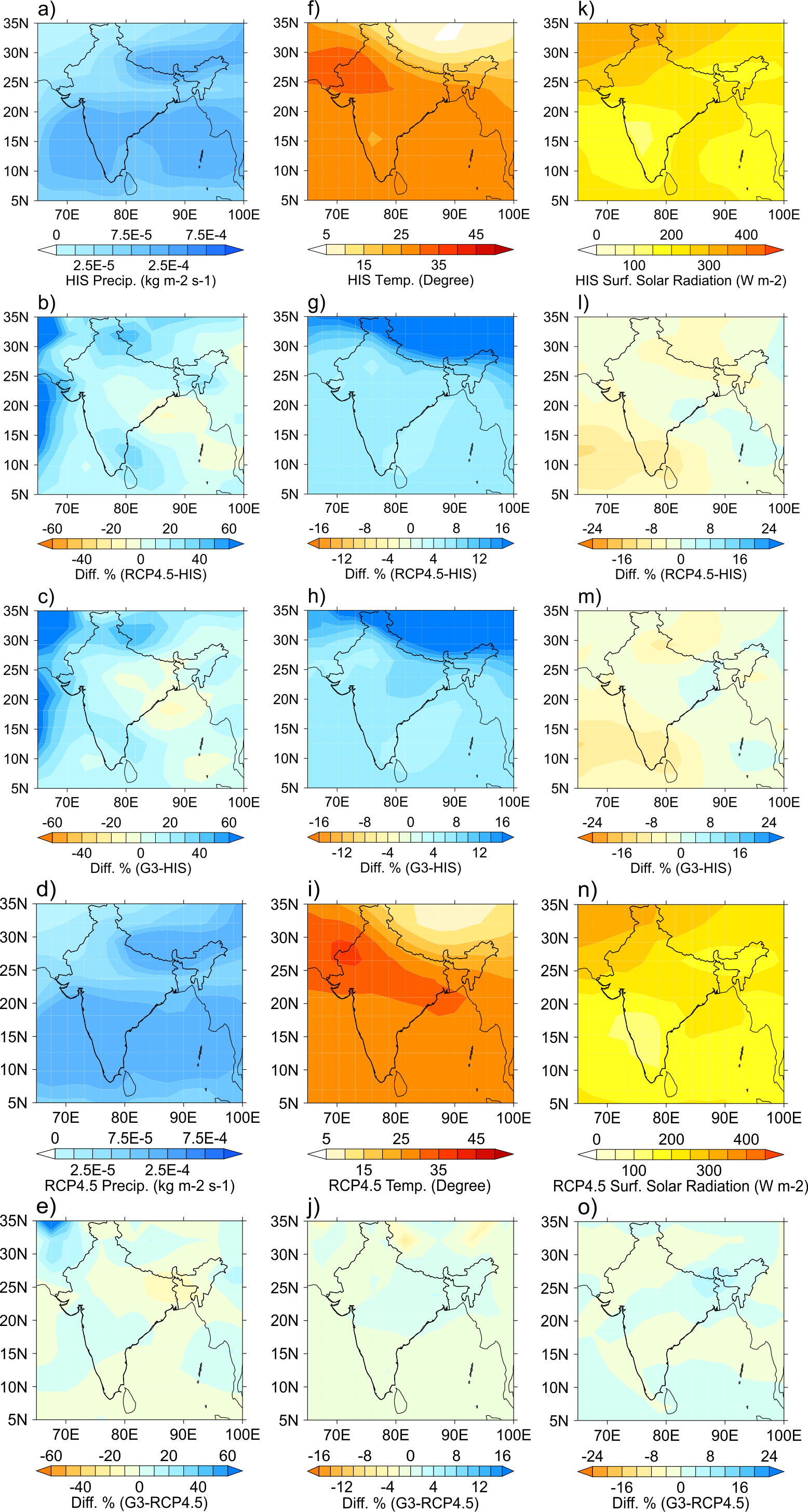

Supplement: Supplementary file 4 — Figure S3 [file GRL-43-11786-s004.png]

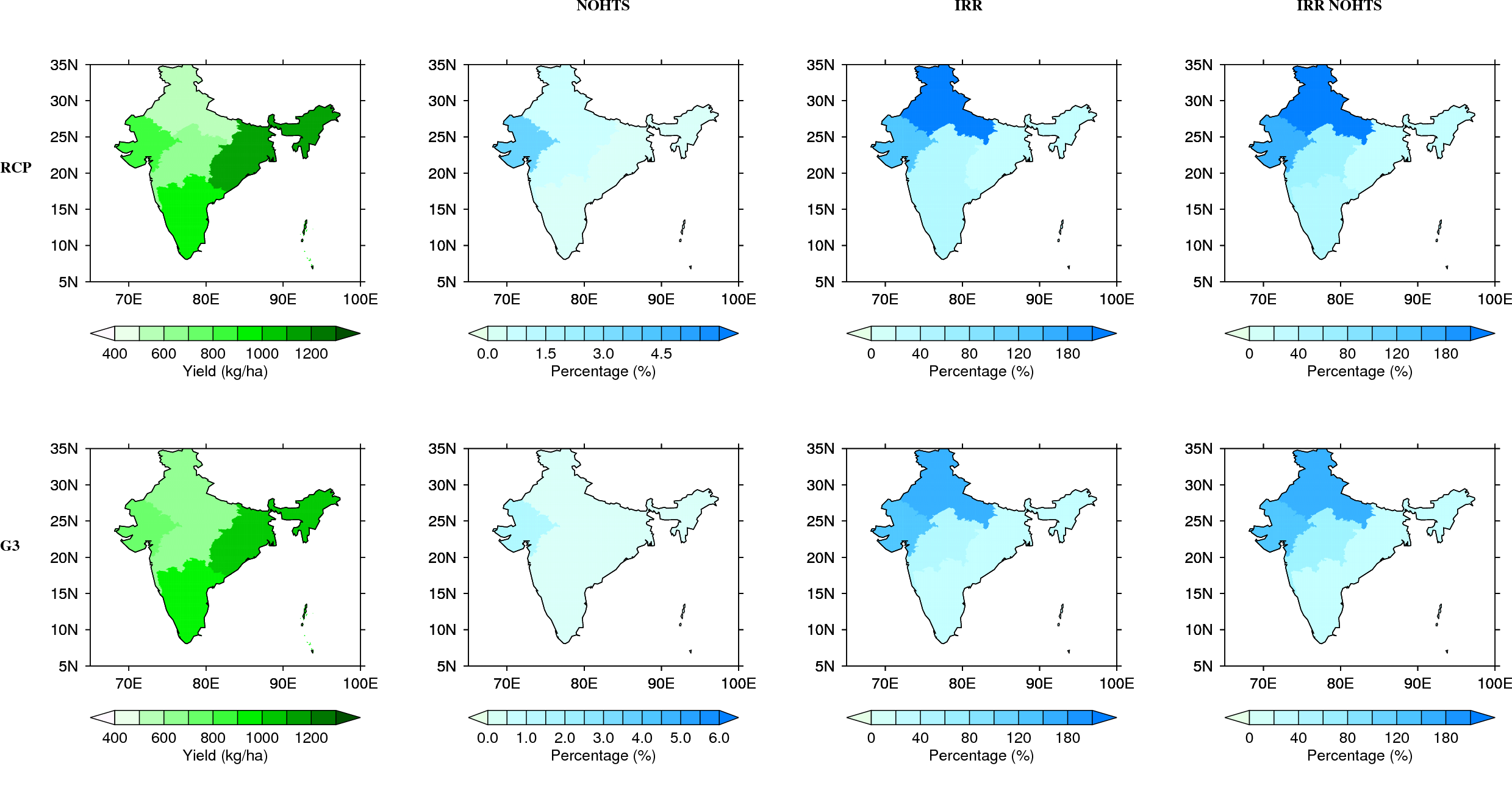

Supplement: Supplementary file 5 — Figure S4 [file GRL-43-11786-s005.png]
